# Rapid establishment of an ICU using anesthesia ventilators during COVID-19 pandemic: lessons learned

**DOI:** 10.1186/s13054-020-03107-x

**Published:** 2020-06-30

**Authors:** Amanda S. Xi, Marvin G. Chang, Edward A. Bittner

**Affiliations:** 1 Division of Critical Care, Department of Anesthesia, Critical Care and Pain Medicine, The Massachusetts General Hospital, Harvard Medical School, Boston, MA USA; 2 Division of Cardiac Anesthesia and Critical Care, Department of Anesthesia, Critical Care and Pain Medicine, The Massachusetts General Hospital, Harvard Medical School, Boston, MA USA

**Keywords:** Ventilator shortage, Anesthesia ventilators, Ventilator deployment, ICU shortage, COVID-19, Coronavirus

We read with great interest the article by Goh et al. [1], and we thought it might be helpful to share our challenging experience with the deployment of anesthesia ventilators in our ICU when faced with a recent ventilator shortage at our institution.

In April 2020, Massachusetts General Hospital saw a significant surge in demand for ICU beds and ventilators due to an influx of COVID-19 patients. We converted our post-anesthesia care units (PACUs) to ICUs and utilized anesthesia ventilators to care for more than 40 COVID-19 patients. While this experience posed challenges, important lessons were learned.

These PACUs turned ICUs required dedicated anesthesia providers to serve as respiratory therapists to manage and troubleshoot the anesthesia ventilators which are functionally different from standard ICU ventilators. Due to the circle system design of anesthesia ventilators and a lack of circuit water traps, higher gas flow rates and frequent circuit changes were required thereby increasing providers’ risk of exposure. Lack of humidification in the circuit resulted in thicker airway secretions and airway obstruction. The heat and moisture exchanger filters which are utilized on anesthesia ventilators clogged easily leading to frequent high airway pressure alarms. Special attention to the position of the side-stream gas analyzer was also required when the patient was in the prone position because condensation quickly accumulated in the water trap when the circuit connection was in the dependent position. In addition, safe deployment of anesthesia ventilators required integration with the centralized alarm system.

The anesthesia ventilators utilized in our ICUs did not have a volume assist-control ventilation mode which led to dyssynchrony, barotrauma, desaturation, auto positive end-expiratory pressure (auto-PEEP), and hemodynamic instability when volume control was employed without the patient paralyzed. Thus, pressure support (PS) mode became the primary mode of ventilation utilized. An important requirement for use of the PS mode was setting a backup respiratory rate to avoid complications such as hypoventilation when sedatives and/or paralysis were administered. Avoiding adverse events from untimely response to alarms required anesthesia providers to maintain vigilance in the setting of increased alarm fatigue; therefore, a greater than expected staffing model was required to maintain patient safety.

Despite these challenges, the use of anesthesia ventilators provided a unique opportunity to use volatile anesthetics, which have been associated with faster extubation times and opioid-sparing effects [2].

While anesthesia ventilators expanded our capacity, we rapidly learned their limitations and potential benefits for long-term use.

## Authors’ response

Goh KJ, Wong J, Tien JC, Ng SY, Duu Wen S, Phua GC, Leong CK

Dear Editor,

We commend Xi et al. for their resilience and innovation in managing the ventilator shortage in their institution and agree with many of the points made. The COVID-19 pandemic has made it necessary for many physicians to implement unconventional strategies to increase access to intensive care, including the use of a single ventilator to ventilate multiple patients [3].

Indeed, there are many challenges associated with the use of anesthetic machines as ventilators. The problems with the circle circuit, side-stream gas analyzer, and need for more frequent changes of the heat and moisture exchanger filters are unique to anesthetic machines. The manpower requirement for a dedicated anesthesiologist to run the machine was mitigated by the reduction in elective surgical workload during the pandemic.

Fortunately, many anesthetic machines are also able to deliver more advanced modes of ventilation, including volume assist-control ventilation, pressure-regulated volume control, and synchronized intermittent mandatory ventilation, reducing the risk of dyssynchrony and barotrauma. The use of an anesthetic machine also facilitates the use of inhalationals, which confers the advantage of a reduction in dependence on intravenous infusions of sedatives during a supply chain disruption [1].

Apart from ventilation, other challenges exist when converting post-anesthetic care units into intensive care units. Central monitoring may not be possible, and a larger number of nursing or medical staff will therefore be required to watch for any clinical changes or observe for alarms. Due to existing infrastructural limitations, restrictions may exist with other modes of organ support. For instance, absence of taps and drainage may preclude the use of intermittent haemodialysis. Intensive care physicians may have to triage patients carefully and assign an appropriate intensive care unit according to the level of organ support that can be safely delivered [4].

Finally, infection prevention measures may be compromised when the patients are cohorted in the post-anesthetic care unit rather than cared for in single rooms. This is an important consideration especially if emergency surgery still needs to take place within the same operating theater complex [5].

Unique challenges exist at each institution depending on its clinical needs and available resources. The best and safest strategy is the one tailored to the individual hospital and current situation.

## Data Availability

Not applicable
